# Identification of Chronic Hypertension in Pregnancy in Three Administrative Data Sources Among Medicaid‐Funded Births in California

**DOI:** 10.1002/pds.70059

**Published:** 2024-12-11

**Authors:** Erin Delker, Rebecca J. Baer, Christina D. Chambers, Gretchen Bandoli

**Affiliations:** ^1^ Department of Pediatrics University of California San Diego San Diego California USA; ^2^ California Preterm Birth Initiative University of California San Francisco San Francisco California USA

**Keywords:** administrative data, chronic hypertension, diagnosis codes, pregnancy

## Abstract

**Purpose:**

Administrative data sources are used to describe the epidemiology of chronic hypertension in pregnancy and its consequences. Differences in identification across sources may affect research estimates. We compared identification of chronic hypertension in birth certificate records, hospital discharge records, and Medi‐Cal claims in the same individuals.

**Methods:**

We used data from 820 140 2016–2020 California Medi‐Cal covered births. We identified chronic hypertension on birth certificates using the prepregnancy hypertension check box and in hospital discharge records and Medi‐Cal claims using ICD codes. We compared the prevalence of chronic hypertension and identified predictors of agreement. We also compared absolute and relative estimates of racial/ethnic disparities in chronic hypertension and associations with neonatal outcomes.

**Results:**

The prevalence of chronic hypertension was 0.7% in birth records, 2.1% in hospital discharge records, and 3.9% in Medi‐Cal claims. There was low to fair agreement between birth certificate records and hospitalization records (kappa = 0.36) and Medi‐Cal claims (kappa = 0.25). Characteristics associated with the worst agreement were eligibility for Women Infant and Children benefits, US‐born, and normal body mass index. Estimates of the relative risk for racial/ethnic disparities and associations with preterm birth and SGA age at delivery were similar across sources. Estimates of risk differences were larger in hospitalization and Medi‐Cal claims data.

**Conclusions:**

Reliance on birth certificate data may contribute to underestimated prevalence estimates and biased causal estimates. Epidemiologic research and public health surveillance of chronic hypertension and its consequences should incorporate data from multiple data sources to improve validity of estimates.

**Summary**

- The prevalence of chronic hypertension was under ascertained in birth records compared with hospitalization records and Medi‐Cal claims for 2016–2020 California births.
- Agreement between birth certificate records and hospitalization and Medi‐Cal claims was low to fair.
- Characteristics associated with the worst agreement were eligibility for Women Infant and Children benefits, US‐born, and normal body mass index.
- Estimates of racial and ethnic disparities in chronic hypertension were meaningfully different across data sources when estimated on the absolute, but not the relative, scale.
- The adjusted risk ratios for the association between chronic hypertension and small‐for‐gestational age were similar across data sources, though the adjusted risk differences varied. The adjusted risk ratios and risk differences for preterm birth were largest in hospitalization data.

## 1 Introduction

Chronic hypertension in pregnancy is defined as high blood pressure (systolic > 140 or diastolic > 90 mmHg) diagnosed or present before pregnancy or before 20 weeks gestation. This definition includes both essential hypertension (~90% of cases) and secondary hypertension. Essential hypertension refers to high blood pressure of unknown cause, whereas, secondary hypertension refers to high blood pressure caused by a medical comorbidity such as renal or heart disease [1].

Chronic hypertension complicates between 1%–3% of US pregnancies [1, 2, 3, 4, 5, 6, 7], with large inequalities by race/ethnicity (highest among Black and American Indian or Alaska Native individuals) [2, 7] and rurality (highest in rural regions) [6]. The prevalence has doubled in the past decade and is expected to rise with increases in maternal age [2, 8]. These trends are concerning due to strong associations with adverse maternal and neonatal outcomes. For example, chronic hypertension is associated with a two‐to‐three‐fold increased risk of preterm birth and having a small‐for‐gestational age (SGA) baby [9, 10].

Monitoring trends in chronic hypertension and its consequences requires valid measurement. Prevalence estimates have been mostly derived from administrative data sources, specifically birth certificate records and hospital discharge records, which allow examination of large population‐based samples. In birth certificate records, chronic hypertension is coded with a prepregnancy checkbox. In hospital discharge records, it is identified by International Classification of Disease (ICD) codes. These codes include pregnancy‐specific codes (e.g., pre‐existing hypertension complicating pregnancy, childbirth and the puerperium) and nonpregnancy specific codes. There is evidence that chronic hypertension is under ascertained in birth [11, 12, 13, 14] and hospital discharge records [11, 12, 15, 16, 17], which may contribute to heterogeneity in estimates of prevalence, disparities, and associations with neonatal outcomes across sources.

To examine the occurrence and impact of different capture between sources, we leveraged California population‐based data with linked birth certificate records, hospital discharge data, and Medi‐Cal inpatient and outpatient claims. We examined the agreement of chronic hypertension classification between sources and identified factors associated with agreement. Also, we compared estimates of racial/ethnic disparities and associations with neonatal outcomes using different data sources.

## 2 Methods

### 2.1 Data Source and Study Sample

The Study of Outcomes of Mothers and Infants (SOMI) is a cohort derived from all California birth records. Birth and fetal death certificates maintained by California Vital Statistics were linked to hospital discharge, emergency department, and ambulatory surgery records maintained by the California Department of Health Care Access and Information (HCAI). The linkage was probabilistic, pairing records based on a match score that included birth hospital, date of birth, sex, zip code, race/ethnicity, and birth characteristics similarly recorded in hospital discharge records and birth certificates (e.g., cesarean delivery) [18, 19]. The linked HCAI data included diagnoses and procedure codes based on the ICD codes for the year before and after birth for the person giving birth and for the year after birth for the baby. In a subset of Medi‐Cal covered deliveries, birth records were individually linked by state file number to Medi‐Cal inpatient, outpatient, and pharmacy claims maintained by the California Department of Health Care Services (DHCS). The linked DHCS data included ICD diagnosis codes for the 90 days prior to the last menstrual period through the end of gestation. The study was approved by the Committee for the Protection of Human Subjects within the Health and Human Services Agency of the State of California, as well as the University of California San Diego Human Research Protections Program.

**FIGURE 1:**
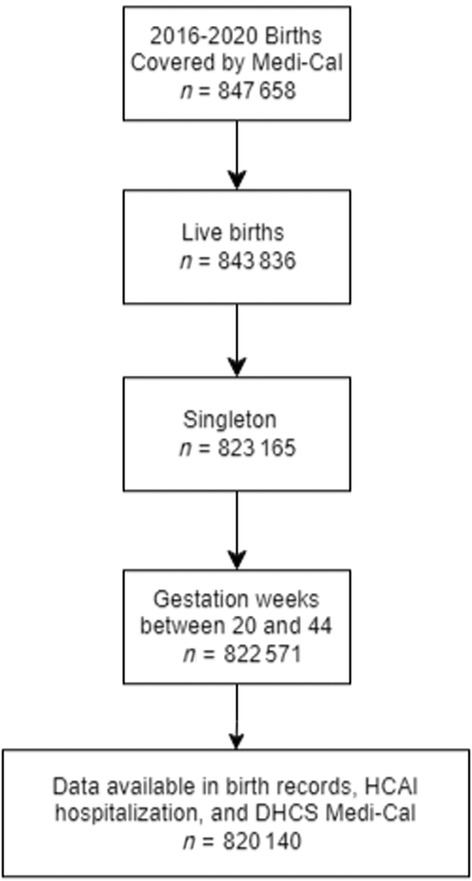
Selection of analytic sample.

We included a subset of 2016–2020 singleton live births where linkage between birth certificates, hospitalization data, and Medi‐Cal data was possible, the payer for delivery was Medi‐Cal, and length of gestation was between 20 and 44 weeks, yielding a sample of 820140 births (Figure 1). Individuals with missing data for payer for delivery or gestational age were not included in the sample.

### 2.2 Measurement

We identified chronic hypertension in three data sources. On birth certificates, we identified chronic hypertension using the prepregnancy hypertension check box. Per the CDC Center for Health Statistics facility worksheet for live birth certificates, the person completing the birth certificate is instructed that if elevation of blood pressure (i.e., hypertension) is present, to “check either prepregnancy or gestational, do not check both.” [20]. In hospital discharge and Medi‐Cal records, we identified chronic hypertension by ICD‐10 codes for the period 90 days before the last menstrual period through the delivery. A list of ICD codes used is in Table S1.

Demographic characteristics for the person giving birth were identified from birth records. These were maternal age at delivery (years), self‐reported race/ethnicity (non‐Hispanic White, Hispanic, Black, American Indian or Alaska Native, Hawaiian or Pacific Islander, multiple race, or other), maternal education (< 12 years, 12 years, > 12 years, missing), Special Supplemental Nutrition Program for Women, Infants, and Children program (WIC) participation, birth country (grouped as United States, Mexico, other), and parity (grouped as nulliparous vs. multiparous). Body mass index was calculated from prepregnancy weight and height on the birth certificate records and categorized as underweight (< 18.5 kg/m^2^), normal weight (18.5–24.9 kg/m^2^), overweight (25.0–29.9 kg/m^2^), and obese (≥ 30 kg/m^2^) [21].

Birth outcomes considered were SGA (birth weight < 10th percentile for gestational age and sex) [22] and preterm birth. Birth weight, infant sex, and gestational age were reported on the birth certificate records. We classified births at < 37 completed weeks gestation as preterm.

### 2.3 Statistical Analysis

We estimated the prevalence of chronic hypertension by source in the full sample and stratified by demographic characteristics. We used birth certificate records, hospitalization discharge records, and Medi‐Cal claims to measure chronic hypertension.

To quantify agreement in the identification of chronic hypertension across sources, we examined pairwise cross‐tabulations and estimated Cohen's Kappa statistics. Kappa values range from −1 to 1 and are interpreted as: ≤ 0 no agreement, 0.01–0.20 none to slight agreement, 0.21–0.40 fair agreement, 0.41–0.60 moderate agreement, 0.61–0.80 substantial agreement, and 0.81–1.00 almost perfect agreement [23].

Next, we estimated racial/ethnic disparities in the prevalence of chronic hypertension. We selected the non‐Hispanic group as the reference category. We used a general linear model with a Poisson distribution and log link function to estimate risk ratios and an identity link function to estimate risk differences [24].

To understand how differential classification of chronic hypertension across sources may affect estimates of the causal effect on preterm birth and SGA, we used a general linear model with a Poisson distribution and log link function to estimate unadjusted and adjusted risk ratios for both outcomes. Then, we used an identity link function to estimate unadjusted and adjusted risk differences. The adjusted models included age at delivery for the person giving birth, education, race/ethnicity, WIC participation, nulliparity, and nativity (Mexico‐born, other foreign born, US born).

In post hoc analyses, we examined factors related to low ascertainment of chronic hypertension in birth certificate records. We selected a group of 16 563 individuals classified as having chronic hypertension in both hospitalization records and Medi‐Cal, then modeled the probability of chronic hypertension being classified in the birth records. Predictor variables were analyzed in separate binary Poisson regression models and included age at delivery, education, race/ethnicity, WIC participation, nulliparity, nativity, and prepregnancy body mass index.

Finally, we conducted a sensitivity analysis examining the frequency of having a diagnosis of both prepregnancy and gestational hypertensive disorders in the hospitalization records and re‐ran analyses using a variable that excluded these cases from the prepregnancy hypertension group. The rationale for this was to improve comparability with birth certificate record data collection, where these variables were mutually exclusive by design because the person filling the form was instructed to select either prepregnancy hypertension or gestational hypertension, depending on when the hypertension started.

## 3 Results

There were 820 140 individuals in the sample. The average age of the person giving birth was 28.2 (SD = 6.0) years with 18% over 34. Most identified as Hispanic (68.3%), participated in WIC (76.4%), had at least a high school level education (68.9%), and were born in the US (57.4%). About one‐third (31.1%) were nulliparous and about two‐thirds were overweight (28.5%) or obese (30.3%) (Table 1).

**TABLE 1:** Demographic characteristics recorded on the birth certificate for Medi‐Cal covered births in California, 2016–2020 (*n* = 820 140).

Characteristic of Person giving birth	Mean (SD) or % (*N*)
Person giving birth age, years	28.2 (SD = 6.0)
< 18 years	1.9 (15523)
18–34 years	80.1 (657183)
> 34 years	18.0 (147419)
Missing	< 0.01 (15)
Race/ethnicity	
American Indian or Alaska Native	0.4 (3314)
Asian	6.6 (53820)
Black	5.9 (48572)
Hawaiian or Pacific Islander	0.4 (3257)
Hispanic	68.3 (560045)
Non‐Hispanic White	14.2 (116020)
Two or more race	1.8 (14973)
Other or missing	2.5 (20138)
WIC	
Yes	76.4 (626308)
No	23.0 (188740)
Missing	0.6 (5092)
Maternal education	
Less than 12 years	25.8 (211784)
12 or more years	68.9 (565179)
Missing	5.3 (43177)
Country of birth	
United States	57.4 (470499)
Mexico	26.2 (215009)
Other	16.4 (134632)
Nulliparous	31.1 (255207)
Pre‐pregnancy Body Mass Index	
Underweight	3.1 (25600)
Normal	35.1 (288070)
Overweight	28.5 (233933)
Obese	30.3 (248725)
Missing	2.9 (23812)

Of the births, 5681 (0.7%) had chronic hypertension identified in birth records, 17 296 (2.1%) in hospital discharge records, and 31 988 (3.9%) in Medi‐Cal (Table 2). When using any data source, the prevalence was 4.1%. The subgroups with the highest prevalence included individuals > 34 years (range 1.5%–7.3%), Black individuals (1.7%–8.3%), and those born in the US (0.8%–4.7%) (Table 2).

**TABLE 2:** Prevalence of chronic hypertension according to birth certificate records, hospitalization records, and inpatient and outpatient claims for the full sample and by maternal characteristics (*n* = 820 140).

Characteristic	Chronic hypertension prevalence % (*N*)		
	Birth certificate	HCAI hospitalization records	DHCS Medi‐Cal inpatient and outpatient claims
Full Sample	0.7 (5681)	2.1 (17296)	3.9 (31988)
Person giving birth age			
< 18 years	0.1 (14)	0.4 (54)	0.8 (131)
18–34 years	0.5 (3501)	1.7 (10952)	3.2 (21 116)
> 34 years	1.5 (2166)	4.3 (6290)	7.3 (10 741)
Missing	0 (0)	0 (0)	0 (0)
Race/ethnicity			
Hispanic	0.6 (3219)	1.8 (9925)	3.4 (19 265)
Non‐Hispanic White	0.8 (968)	2.5 (2838)	4.4 (5065)
Black	1.7 (817)	5.1 (2456)	8.3 (4009)
American Indian/Alaska Native	1.5 (50)	3.6 (118)	5.8 (193)
Hawaiian/Pacific Islander	1.0 (34)	2.7 (88)	5.1 (167)
Asian	0.6 (309)	1.7 (897)	3.0 (1617)
Two or more race	1.1 (162)	3.6 (501)	5.8 (871)
Other or missing	0.6 (122)	2.4 (473)	4.0 (801)
WIC			
Yes	0.7 (4274)	2.1 (13310)	4.0 (24777)
No	0.7 (1365)	2.1 (3860)	3.7 (6985)
Missing	0.8 (42)	2.5 (126)	4.4 (226)
Maternal education			
Less than 12 years	0.6 (1323)	1.9 (3983)	3.5 (7369)
12 or more years	0.7 (4093)	2.2 (12353)	4.1 (22959)
Missing	0.6 (265)	2.2 (960)	3.8 (1660)
Country of birth			
United States	0.8 (3906)	2.6 (12258)	4.7 (22175)
Mexico	0.5 (1101)	1.5 (3191)	2.9 (6227)
Other	0.5 (674)	1.4 (1847)	2.7 (3586)
Nulliparous	0.5 (1381)	1.5 (3864)	3.0 (7702)
Pre‐pregnancy body mass index			
Underweight	0.2 (49)	0.6 (145)	1.2 (294)
Normal	0.3 (726)	0.9 (2449)	1.7 (4786)
Overweight	0.5 (1187)	1.6 (3684)	3.0 (6989)
Obese	1.4 (3578)	4.2 (10412)	7.6 (18880)
Missing	0.6 (141)	2.5 (606)	4.4 (1039)

Agreement between birth certificates and hospitalization records (kappa = 0.36) and Medi‐Cal (kappa = 0.25) was low to fair. Only 24.2% of the chronic hypertension cases in hospitalization records and 15.5% in Medi‐Cal were recorded in the birth record. Agreement between hospitalization records and Medi Cal claims was better (kappa = 0.66), though only 51.8% of chronic hypertension cases identified in Medi‐Cal inpatient and outpatient claims were identified in hospitalization records. Of cases identified in hospitalization records, 95.8% were also identified in Medi‐Cal (Table 3).

**TABLE 3:** Agreement in the classification of chronic hypertension across birth certificate records, hospitalization records, and inpatient and outpatient claims (*n* = 820 140).

	Chronic hypertension prevalence, *n* (%)	Agreement between sources		
		[1]	[2]	[3]
[1] Birth certificate	5681 (0.69)	—	4177 (73.5%) kappa = 0.36	4946 (87.1%) kappa = 0.25
[2] HCAI hospitalization records	17 296 (2.11)	4177 (24.2%)	—	16 563 (95.8%) kappa = 0.66
[3] DHCS Medi‐Cal inpatient and outpatient claims	31 988 (3.90)	4946 (15.5%)	116 563 (51.8%)	—
Any data source	33 359 (4.07)			

*Note:* Percentages displayed in “Agreement between sources” columns are row percentages. For example, Of the 5681 chronic hypertension cases identified in birth certificate records, 4177 were identified in HCAI hospitalization data and 4946 were identified in DHCS Medi‐Cal claims.

Abbreviations: DHCS = California Department of Health Care Services; HCAI = California Department of Health Care Access and Information.

When using the birth records to classify chronic hypertension, the risk ratios comparing each racial and ethnic group to the non‐Hispanic White group (reference) were 0.7 (95%CI: 0.6 to 0.7) for Hispanic, 2.0 (95%CI: 1.8 to 2.2) for Black, 1.8 (95%CI: 1.4 to 2.4) for American Indian or Alaska Native, 1.3 (95%CI: 0.9 to 1.8) for Hawaiian or Pacific Islander, 0.7(95%CI: 0.6 to 0.8) for Asian, and 1.3 (95%CI: 1.1 to 1.5) for two or more races. These estimates were similar using hospitalization or Medi‐Cal records, except for the American Indian or Alaska Native group, which was attenuated to 1.5 (95%CI: 1.2 to 1.7) in hospitalization records, and 1.3 (95%CI: 1.2 to 1.5) in Medi‐Cal (Table 4).

Conversely, risk differences for chronic hypertension by racial and ethnic group varied by data source. For example, the unadjusted risk difference for chronic hypertension comparing Black to non‐Hispanic White individuals was 0.9 per 100 persons using birth records, 2.6 per 100 persons using hospitalization data, and 3.9 per 100 persons using Medi‐Cal claims (Table 4).

The prevalence of SGA age ranged from 12.4%–13.4% in the chronic hypertension group. The adjusted risk ratios were the same in the birth records (RR: 1.4, 95%CI: 1.3–1.5) as in hospitalization records (RR: 1.4, 95%CI: 1.4–1.5) and Medi‐Cal (RR: 1.4, 95% CI: 1.3–1.4) Table 5. The adjusted risk difference was largest in birth records (RD: 3.9 per 100 persons, 95%CI: 3.0–4.8) compared with hospitalization records (RD: 3.7 per 100 persons, 95%CI: 3.2–4.1) and Medi‐Cal (RD: 3.2 per 100 persons, 95%CI: 2.8–3.5) (Table 5).

**TABLE 4:** Relative and absolute racial and ethnic disparities according to birth certificate records, hospitalization records, and inpatient and outpatient claims (*n* = 820 140).

Race/ethnicity	Birth Records			HCAI hospitalization records			DHCS Medi‐Cal Claims		
	%	RR	RD	%	RR	RD	%	RR	RD
Non‐Hispanic White	0.8	ref	ref	2.5	ref	ref	4.4	ref	ref
Hispanic	0.6	0.7 (0.6, 0.7)	−0.3 (−0.3, −0.2)	1.8	0.7 (0.7, 0.8)	−0.7 (−0.8, −0.6)	3.4	0.8 (0.8, 0.8)	−0.9 (−1.1, −0.8)
Black	1.7	2.0 (1.8, 2.2)	0.9 (0.7, 1)	5.1	2.1 (2, 2.2)	2.6 (2.4, 2.8)	8.3	1.9 (1.8, 2)	3.9 (3.6, 4.2)
American Indian/Alaska Native	1.5	1.8 (1.4, 2.4)	0.7 (0.3, 1.1)	3.6	1.5 (1.2, 1.7)	1.1 (0.5, 1.8)	5.8	1.3 (1.2, 1.5)	1.5 (0.7, 2.3)
Hawaiian/Pacific Islander	1.0	1.3 (0.9, 1.8)	0.2 (−0.1, 0.6)	2.7	1.1 (0.9, 1.4)	0.3 (−0.3, 0.8)	5.1	1.2 (1, 1.4)	0.8 (0, 1.5)
Asian	0.6	0.7 (0.6, 0.8)	−0.3 (−0.3, −0.2)	1.7	0.7 (0.6, 0.7)	−0.8 (−0.9, −0.6)	3.0	0.7 (0.7, 0.7)	−1.4 (−1.6, −1.2)
Two or more race	1.1	1.3 (1.1, 1.5)	0.3 (0.1, 0.4)	3.4	1.4 (1.2, 1.5)	0.9 (0.6, 1.2)	5.8	1.3 (1.2, 1.4)	1.5 (1.1, 1.8)
Other or missing	0.6	0.7 (0.6, 0.9)	−0.2 (−0.4, −0.1)	2.4	1 (0.9, 1.1)	−0.1 (−0.3, 0.1)	4.0	0.9 (0.8, 1)	−0.4 (−0.7, −0.1)

*Note:* Risk differences are expressed per 100 persons. Estimates are unadjusted.

Abbreviations: CI = confidence interval; RD = risk difference; RR = risk ratio.

**TABLE 5:** Frequencies, relative risks, and risk differences for small‐for‐gestational age delivery and preterm birth when classifying chronic hypertension in birth certificate records, hospitalization records, and inpatient and outpatient claims (*n* = 820 140).

Outcome	Measure of chronic hypertension	Chronic hypertension		Relative risk (95% CI)		Risk difference (95% CI)	
		N	Cases (%)	Unadjusted	Adjusted	Unadjusted	Adjusted
Small for gestational age at delivery	Birth records	5681	462 (13.4)	1.4 (1.4, 1.5)	1.4 (1.3, 1.5)	4.2 (3.3, 5)	3.9 (3, 4.8)
	HCAI hospitalization	17 296	2243 (13.0)	1.4 (1.4, 1.5)	1.4 (1.4, 1.5)	3.8 (3.3, 4.3)	3.7 (3.2, 4.1)
	DHCS Medi‐Cal	31 988	3963 (12.4)	1.4 (1.3, 1.4)	1.4 (1.3, 1.4)	3.3 (2.9, 3.7)	3.2 (2.8, 3.5)
Preterm birth	Birth records	5681	1284 (22.6)	2.9 (2.8, 3.1)	2.5 (2.4, 2.6)	14.9 (13.8, 16)	13.7 (12.7, 14.8)
	HCAI hospitalization	17 296	4180 (24.2)	3.3 (3.2, 3.3)	2.9 (2.8, 2.9)	16.8 (16.1, 17.4)	15.7 (15.1, 16.3)
	DHCS Medi‐Cal	31 988	7079 (22.1)	3.1 (3, 3.1)	2.8 (2.7, 2.8)	14.9 (14.5, 15.4)	14.1 (13.6, 14.6)

*Note:* Risk differences are expressed per 100 persons. Adjusted models include person giving birth age, education, race/ethnicity, WIC, parity, and nativity.

Abbreviations: CI = confidence interval; RD = risk difference; RR = risk ratio.

The prevalence of preterm birth ranged from 22.1% to 24.2% in the chronic hypertension group. The adjusted risk ratios were 2.5 (95%CI: 2.4–2.6) in birth records, 2.9 (95%CI: 2.8–2.9) in hospitalization records, and 2.8 (95% CI: 2.7–2.8) in Medi‐Cal. The adjusted risk difference estimate was largest in hospitalization records (RD: 15.7 per 100 persons, 95%CI: 15.1–16.3), followed by Medi‐Cal (RD: 14.1 per 100 persons, 95%CI: 13.6–14.6) and birth records (RD: 13.7 per 100 persons, 95%CI: 12.7–14.8) (Table 5).

In post hoc analyses, we examined identification of chronic hypertension in birth certificate records among 16 563 individuals where chronic hypertension was identified in both hospitalization and Medi‐Cal records. Variables significantly associated with agreement in chronic hypertension classification on birth certificates were race/ethnicity (lowest among Hispanic individuals, 23.8%), WIC eligibility (lowest among individuals eligible for WIC, 23.9%), birth country (lowest among US‐born individuals, 24.4%), nulliparity (lowest among multiparous individuals, 24.5%), and prepregnancy body mass index (lowest among individuals with normal body mass index, 20.3%) (Table S2).

In sensitivity analyses, we updated the coding of our HCAI hospitalization record prepregnancy hypertension definition to exclude any individuals with additional diagnoses of gestational hypertension or preeclampsia as zeros, to be consistent with the mutually exclusive nature of how these variables were recorded on the birth record. The prevalence estimate dropped from 2.3% to 1.4% and kappa estimate of agreement changed from 0.35 to 0.32 (Table S3).

## 4 Discussion

We examined agreement in identifying chronic hypertension in pregnancy across birth records, hospitalization records, and Medi‐Cal claims. Specifically, we compared differences in prevalence estimates, racial/ethnic disparities, and associations with neonatal outcomes.

We estimated the 2016–2020 California prevalence of chronic hypertension as 0.7% in birth records, 2.1% in hospitalization records, 3.9% in Medi‐Cal claims, and 4.1% using any source. A study examining US state‐level variation in hypertensive disorders of pregnancy using birth certificate data reported a similar prevalence for California around 0.9% [5]. It has been proposed that birth certificates are not a valid data source for maternal risk factors and that reliance on birth certificate data alone will result in underestimates of the prevalence of maternal conditions [11, 12, 13, 14, 25]. The coding accuracy for hypertensive disorders varies by state. For example, in New York, the sensitivity for hypertensive disorders on birth records was reported as 39% compared with 76% in Vermont [26]. Our study adds that chronic hypertension, specifically, is under ascertained on birth records in California. For example, we reported that the proportion of people that had chronic hypertension identified on both hospitalization and Medi‐Cal records with chronic hypertension also identified on birth records was 25%, meaning 75% of individuals that were flagged as having chronic hypertension in both other sources (a proxy for having chronic hypertension) were missed on the birth certificate. Our findings also add that the under ascertainment of chronic hypertension on birth certificate records in California is nonrandom. For example, we reported that Black individuals, people born outside of Mexico and the US, people not eligible for WIC benefits, and overweight and obese people were more likely to have chronic hypertension checked on the birth record in agreement with other sources. More information on the training, priorities, and data access of birth certificate coders is needed to understand these findings. Birth certificate data alone should not be used to characterize the epidemiology of chronic hypertension.

The California Department of Health (CDPH) defines chronic hypertension using ICD codes in inpatient delivery hospitalization records. For the years 2016 to 2020, corresponding to our study period, the CDPH estimated annual prevalence estimates between 2.2% and 2.3%, which matched our estimate of 2.1% derived from hospitalization records [27]. Of note, we included data from all hospitalizations in the 90 days prior to the last menstrual period through a year after birth, whereas the CDPH estimates only considered the delivery encounter. This may suggest that inclusion of data from hospitalizations outside of the delivery encounter do not meaningfully change the identification of chronic hypertension. However, our prevalence estimates may have been larger if we considered a longer prepregnancy “look back” period.

The data that yielded the highest prevalence estimate for chronic hypertension was Medi‐Cal claims. This was expected because Medi‐Cal includes an individual's prenatal records, where pregnant individuals would've been screened for hypertension prior to pregnancy. Still, our estimate of 3.9% from Medi‐Cal underestimates a 2009–2014 Kaiser Permanente Southern California estimate of 4.3%, which used in‐office BP measurements [28]. Although the latter sample is not directly comparable because Kaiser Permanente Southern California is a distinct population, it may be that data sources with more detailed data from healthcare encounters, such as in‐office blood pressure readings, result in higher prevalence estimates [29]. It is also worth noting that estimates of self‐reported prepregnancy hypertension from the California Maternal and Infant Health Assessment (MIHA) Survey for 2019–2021 was 3.5% (95% CI: 3.1, 2.8) and for 2016–2018 was 3.1% (95% CI: 2.8, 3.4%) [30].

Across sources, the prevalence of chronic hypertension was relatively higher using the “any source” approach (4.1%) and was similar to the use of DHCS Medi‐Cal claims alone. The majority of cases that were identified in either birth records or HCAI hospitalization data were also flagged as having chronic hypertension in Medi‐Cal claims. This was expected considering the hospitalization data for the birth encounter should be the same in both HCAI and Medi‐Cal sources. Lower than expected kappa statistics for agreement between HCAI and DHCS Medi‐Cal were due to about 52% of cases that were identified in DHCS but missed in the hospitalization records.

Our sample was restricted to an insured, Medi‐Cal covered population. Our estimates from Medi‐Cal claims relied on ICD codes, which require engagement with care and adequate prenatal care visits, or in other words, the opportunity to be diagnosed. This may miss individuals from marginalized racial/ethnic groups, those living in rural areas, or individuals with other barriers to accessing care. Also, there are normative declines in blood pressure during the first trimester. So, individuals with undiagnosed chronic hypertension that seek care early in pregnancy may be missed because their measured blood pressure readings fall below clinical thresholds for diagnosis at that point in gestation [31]. These factors may have contributed to an underestimation of the true prevalence, which cannot be determined from these data. Future research that additionally includes primary data collection to directly measure hypertension prior to pregnancy is needed.

Our estimates of chronic hypertension by race/ethnicity show that Black and American Indian or Alaska Native individuals had a higher prevalence compared with other groups, consistent with prior studies [2, 5, 7]. Using the non‐Hispanic White group as the reference, we estimated racial/ethnic disparities on the relative (risk ratios) and absolute (risk differences) scales because inferences drawn may be scale dependent [32]. Absolute measures provide more information regarding the population burden of disparities between groups. Relative measures more easily allow for the comparison of disparities across health outcomes, though don't consider absolute rates of disease in each group or the population share of the group [32, 33, 34]. In our study, the risk ratios were generally similar across data sources. The risk differences were similar in hospitalization records and Medi‐Cal data claims, but smaller in birth records. The small risk differences from the birth records were driven by lower ascertainment of chronic hypertension across all groups. While no prior studies have compared estimates of race/ethnic disparities for chronic hypertension, specifically, there have been similar studies on related outcomes. For example, using data from California and Michigan, researchers observed a larger Black‐White disparity in severe maternal morbidity when using hospitalization versus birth certificate data [35]. A study using data from Southern California hospitals compared the ratio of pre‐eclampsia incidence estimates in birth certificates to hospitalization data and reported lower ratios among Asian (0.40) and Hispanic (0.39) individuals compared to Black (0.46), and non‐Hispanic (0.50) individuals [36]. The second study also showed differences in the ratio of incidence rates by maternal education and insurance, which were not explored in the current study but should be examined in future research.

We reported that chronic hypertension was associated with 1.4 times higher risk for SGA age at delivery and 2–3 times higher risk of preterm birth in the three data sources, similar to estimates reported in review papers [9, 10]. We had expected that associations would be larger when estimated using birth records, assuming that chronic hypertension may have been more likely to be checked on the birth record in pregnancies that resulted in an adverse outcome. However, the relative risks for SGA were the same across sources and the relative risk for preterm birth was smaller in birth records. Like estimates of racial/ethnic disparities, associations with neonatal outcomes were more sensitive to the data source when estimated on the absolute scale.

Limitations should be considered. First, we analyzed data from three administrative data sources and did not include primary measurements of blood pressure and thus have no “gold standard.” Second, we did not investigate differences in classification by specific ICD codes. It is possible that agreement across sources varied by use of pregnancy versus nonpregnancy codes. Third, we evaluated associations between chronic hypertension and SGA and preterm birth, and did not consider potential misclassification of the study outcomes. Also, we restricted our sample to individuals where the delivery was covered by Medi‐Cal. Findings from this group may not be generalizable to all California pregnancies or pregnancies in other places. Finally, we did not have the data available to additionally identify prepregnancy hypertension through use of antihypertensive medication pharmacy claims, an approach that has been used in prior research. We encourage others with access to pharmacy claims data to replicate our research and estimate the impact of additionally adding claims data to the prepregnancy hypertension definition.

Despite limitations, we examined agreement of chronic hypertension using a large sample size and three linked data sources. We conclude that researchers should take caution in relying on birth records when studying chronic hypertension. Our research shows that capture of chronic hypertension on birth records is poor and this misclassification may vary by sociodemographic and clinical factors. Therefore, reliance on these data may contribute to underestimated prevalence estimates and biased causal estimates. Epidemiologic research and public health surveillance of chronic hypertension and its consequences should incorporate data from multiple data sources to improve validity of estimates.

### Plain Language Summary

High blood pressure before pregnancy increases the risk of bad pregnancy outcomes. We often study people with high blood pressure before pregnancy using data from administrative files. The way we identify cases differs by data source, which means that we may get different answers to our research questions depending on the data we use. Here, we identified people with high blood pressure during pregnancy using three data sources: birth certificates, hospitalization records, and Medi‐Cal claims. We used a sample of 820 140 California births and ran three main analyses in each of the data sources. First, we estimated the proportion of individuals with high blood pressure before pregnancy. Second, we estimated racial and ethnic differences in this proportion. Third, we compared the risk of preterm birth and having a small baby according to whether someone had high blood pressure before pregnancy. Our findings show that the proportion of people with high blood pressure before pregnancy was lowest when using birth certificate records compared with hospitalization and Medi‐Cal claims data sources. This difference in identification of cases was not random and resulted in different estimates of the racial and ethnic disparities and associations with neonatal outcomes.

## Ethics Statement

The Study of Outcomes in Mothers and Infants study and Medi‐Cal data linkage was approved by the Committee for the Protection of Human Subjects within the Health and Human Services Agency of the State of California and the University of California San Diego Human Research Protections Program. Medi‐Cal data was available via a data use agreement (DUA # 22–09‐04) with the California Department of Health Care Services.

## Conflicts of Interest

The authors declare no conflicts of interest.

## Supporting information


**Table S1.**. International Classification of Diseases 10th Edition (ICD‐10) codes used to identify chronic hypertension in HCAI and DHCS files.
**Table S2**. Predictors of chronic hypertension classified in birth records when chronic hypertension was identified in both HCAI hospitalization records and DHCS medical claims (*n* = 16 563).
**Table S3**. Agreement in the classification of chronic hypertension between vital statistics and HCAI hospitalization records when excluding individuals with additional diagnoses of gestational hypertension or preeclampsia (*n* = 820 140).
