# Supplementary material for: Identification of Chronic Hypertension in Pregnancy in Three Administrative Data Sources Among Medicaid‐Funded Births in California
Source: Pharmacoepidemiol Drug Saf. 2024 Dec 11;33(12):e70059. doi: 10.1002/pds.70059 (PMC11634560; doi:10.1002/pds.70059)
Supplement: Supplementary file 1 — Table S1.. International Classification of Diseases 10th Edition (ICD‐10) codes used to identify chronic hypertension in HCAI and DHCS files. Table S2. Predictors of chronic hypertension classified in birth records when chronic hypertension was identified in both HCAI hospitalization records and DHCS medical claims (n = 16 563). Table S3. Agreement in the classification of chronic hypertension between vital statistics and HCAI hospitalization records when excluding individuals with additional diagnoses of gestational hypertension or preeclampsia (n = 820 140). [file PDS-33-e70059-s001.docx]

Supplementary Table 1. International Classification of Diseases 10th Edition (ICD-10) codes used to identify chronic hypertension in HCAI and DHCS files.

| ICD-10 Code | Description |
| --- | --- |
| Standard codes (in any position) start with… | |
| I10 | Essential (primary) hypertension |
| I11 | Hypertensive heart disease |
| I12 | Hypertensive kidney disease |
| I13 | Hypertensive heart and chronic kidney disease |
| I15 | Secondary hypertension |
| Pregnancy-Related codes (in any position) starting with… | |
| 010.0 | Pre-existing essential hypertension complicating pregnancy, childbirth, and the puerperium |
| O10.1 | Pre-existing hypertensive heart disease complicating pregnancy, childbirth, and the puerperium |
| O10.2 | Pre-existing hypertensive chronic kidney disease complicating pregnancy, childbirth, and the puerperium |
| O10.3 | Pre-existing hypertensive heart and chronic kidney disease complicating pregnancy, childbirth, and the puerperium |
| O10.4 | Pre-existing secondary hypertension complicating pregnancy, childbirth, and the puerperium |
| O10.9 | Unspecified pre-existing hypertension complicating pregnancy, childbirth, and the puerperium |
| O11 | Preeclampsia superimposed on chronic hypertension |

Note: addition of I16 (hypertensive crisis) to the standard I codes in sensitivity analysis did not change estimates. Individuals with and I16 always had at least one other I code in their record.

Supplementary Table 2. Predictors of chronic hypertension classified in birth records when chronic hypertension was identified in both HCAI hospitalization records and DHCS medi-cal claims (n = 16563)

| **Characteristic of Person Giving Birth** | **% (N) Identified In Birth Records** | **Risk Ratios (95% CI)** |
| --- | --- | --- |
| Full sample | 24.6 (4080) |  |
| Person giving birth age |  |  |
| < 18 years | < 11 | ref |
| 18 - 34 years | 22.9 (2389) | 1.9 (0.8, 4.4) |
| > 34 years | 27.6 (1686) | **2.3 (1.0, 5.3)** |
| Race/ethnicity |  |  |
| Hispanic | 23.8 (2259) | 0.9 (0.9, 1.0) |
| Non-Hispanic White | 25.4 (690) | ref |
| Black | 26.9 (631) | **1.1 (1.0, 1.2)** |
| American Indian or Alaska Native | 28.5 (33) | 1.1 (0.8, 1.5) |
| Hawaiian or Pacific Islander | 27.6 (24) | 1.1 (0.8, 1.5) |
| Asian | 26.8 (231) | 1.1 (0.9, 1.2) |
| Two or more race | 24.5 (115) | 1 (0.8, 1.1) |
| Other or missing | 21.1 (97) | 0.8 (0.7, 1) |
| WIC |  |  |
| Yes | 23.9 (3054) | **0.9 (0.8, 0.9)** |
| No | 27.0 (994) | ref |
| Maternal education |  |  |
| Less than 12 years | 24.3 (936) | ref |
| 12 years or more | 24.9 (2937) | 1.0 (1.0, 1.1) |
| Country of birth |  |  |
| United States | 24.4 (2847) | ref |
| Mexico | 24.6 (760) | 1 (0.9, 1.1) |
| Other | 26.5 (473) | **1.1 (1.0, 1.2)** |
| Nulliparous |  |  |
| Yes | 25.0 (911) | 1 (1, 1.1) |
| No | 24.5 (3169) | ref |
| Pre-pregnancy Body Mass Index |  |  |
| Underweight | 22.5 (29) | 1.1 (0.8, 1.5) |
| Normal | 20.3 (468) | ref |
| Overweight | 23.6 (826) | **1.2 (1.1, 1.3)** |
| Obese | 26.4 (2652) | **1.3 (1.2, 1.4)** |

< 11: Data is masked in compliance with Institutional Review Board protocols

Supplementary Table 3. Agreement in the classification of chronic hypertension between vital statistics and HCAI hospitalization records when excluding individuals with additional diagnoses of gestational hypertension or preeclampsia (n = 820,140).

|  | Chronic hypertension prevalence, n (%) | Agreement between sources | |
| --- | --- | --- | --- |
|  |  | [1] | [2] |
| [1] Birth Certificate | 5681 (0.69) | —- | 2755 (48.5%)  kappa = 0.32 |
| [2] HCAI hospitalization records | 11113 (1.36) | 2755 (24.8%) | —- |
| HCAI = California Department of Health Care Access and Information | | | |
